# Suppression subtractive hybridization identified differentially expressed genes in lung adenocarcinoma: *ERGIC3* as a novel lung cancer-related gene

**DOI:** 10.1186/1471-2407-13-44

**Published:** 2013-02-01

**Authors:** Mingsong Wu, Tao Tu, Yunchao Huang, Yi Cao

**Affiliations:** 1 Key Laboratory of Animal Models and Human Disease Mechanism, Kunming Institute of Zoology, Chinese Academy of Sciences, Kunming, 650223, China; 2 Graduate University of the Chinese Academy of Sciences, Beijing, 100049, China; 3 Department of Cell Biology and Genetics, Zunyi Medical College, Zunyi, 563003, China; 4 Department of Anesthesiology, First Affiliated Hospital of Kunming Medical University, Kunming, 650032, China; 5 Department of Thoracic Surgery, Tumor Hospital of Yunnan Province, Kunming, 650106, China

**Keywords:** Lung cancer, cDNA library, Suppression subtractive hybridization, *ERGIC3*, *Erv46*

## Abstract

**Background:**

To understand the carcinogenesis caused by accumulated genetic and epigenetic alterations and seek novel biomarkers for various cancers, studying differentially expressed genes between cancerous and normal tissues is crucial. In the study, two cDNA libraries of lung cancer were constructed and screened for identification of differentially expressed genes.

**Methods:**

Two cDNA libraries of differentially expressed genes were constructed using lung adenocarcinoma tissue and adjacent nonmalignant lung tissue by suppression subtractive hybridization. The data of the cDNA libraries were then analyzed and compared using bioinformatics analysis. Levels of mRNA and protein were measured by quantitative real-time polymerase chain reaction (q-RT-PCR) and western blot respectively, as well as expression and localization of proteins were determined by immunostaining. Gene functions were investigated using proliferation and migration assays after gene silencing and gene over-expression.

**Results:**

Two libraries of differentially expressed genes were obtained. The forward-subtracted library (FSL) and the reverse-subtracted library (RSL) contained 177 and 59 genes, respectively. Bioinformatic analysis demonstrated that these genes were involved in a wide range of cellular functions. The vast majority of these genes were newly identified to be abnormally expressed in lung cancer. In the first stage of the screening for 16 genes, we compared lung cancer tissues with their adjacent non-malignant tissues at the mRNA level, and found six genes (*ERGIC3*, *DDR1*, *HSP90B1*, *SDC1*, *RPSA*, and *LPCAT1*) from the FSL were significantly up-regulated while two genes (*GPX3* and *TIMP3*) from the RSL were significantly down-regulated (*P* < 0.05). The ERGIC3 protein was also over-expressed in lung cancer tissues and cultured cells, and expression of ERGIC3 was correlated with the differentiated degree and histological type of lung cancer. The up-regulation of ERGIC3 could promote cellular migration and proliferation in vitro.

**Conclusions:**

The two libraries of differentially expressed genes may provide the basis for new insights or clues for finding novel lung cancer-related genes; several genes were newly found in lung cancer with *ERGIC3* seeming a novel lung cancer-related gene. ERGIC3 may play an active role in the development and progression of lung cancer.

## Background

Lung cancer is the leading cause of cancer-related death and the global five-year survival rate is only 10% to 15%. Lung cancer is also more variable in its biological behavior and can be divided into two histological groups: small-cell lung cancer (SCLC) and non-small cell lung cancer (NSCLC). NSCLC which accounts for approximately 80% of all lung cancers, includes adenocarcinoma (AC), squamous cell carcinoma (SCC) and large-cell carcinoma. The incidence of adenocarcinoma appears to be increasing globally.

Cancer is the result of the accumulation of genetic and epigenetic alterations, which in turn means there are different profiles of gene expression in various lung cancers [1]. Studying differentially expressed genes between cancer and normal tissues is crucial to understanding carcinogenesis and identifying novel biomarkers for cancer [2,3]. Several technologies are available to obtain profiles of the differentially expressed genes: representational difference analysis, serial analysis of gene expression, oligonucleotide microarrays, or suppression subtractive hybridization (SSH) [4], among others. SSH is a polymerase chain reaction (PCR)-based cDNA subtraction technique that allows selective amplification of target cDNA while simultaneously suppressing non-target cDNA amplification. The cDNA library generated by hybridization and subtraction techniques reduces abundantly expressed housekeeping genes or genes commonly expressed in both control and treated individuals, thereby normalizing the cDNA expression profiles during library construction [4]. As a result, this technique significantly enhances the chances of differentially expressed genes [5]. SSH has been successfully applied to a wide variety of malignant diseases including lung cancer for the generation of cDNA libraries [6-10].

In previous studies, samples were obtained from either culture cells or tissues from different individuals. The inherent problem in this sampling was that the SSH library generated using cultured cells may provide some incorrect information, because genes could have varied or mutated. Similarly, the SSH library constructed using tissues of different individual leads to the problem that the differentially expressed genes in various individuals could not be neutralized during hybridization, and these genes could be incorrectly deemed as being cancer-related. To correct these shortfalls, we used lung AC tissue and its adjacent nonmalignant lung tissue to establish two cDNA libraries by SSH, to obtain more accurate information of differentially expressed genes in lung ACs.

After genome BLAST, 177 up-regulated and 59 down-regulated genes in lung ACs were obtained from the forward-subtracted library (FSL) and the reverse-subtracted library (RSL), respectively. Further bioinformatic analysis demonstrated that these genes were involved in a wide range of cellular functions. The vast majority of these genes were newly identified to be abnormally expressed in lung cancer. Subsequently, we selected 16 differentially expressed genes to investigate their mRNA levels on lung cancer tissue samples as the first stage of screening. According to real-time RT-PCR analysis, *DDR1*, *HSP90B1*, *SDC1*, *RPSA*, *ERGIC3*, and *LPCAT1* were up-regulated significantly in NSCLCs, while *GPX3*, *TIMP3* were down-regulated significantly. ERGIC3 is located in endoplasmic reticulum and Golgi apparatus of NRK cells [11], however, the function of ERGIC3 is unclear in lung cancer. Therefore, expression of ERGIC3 in NSCLCs was further confirmed at the protein level by western blot and immunohistochemistry analysis and we studied the pathophysiological functions of *ERGIC*3.

## Methods

### Patients and tissue samples

The primary tumors and adjacent nonmalignant lung tissues were obtained at the time of surgery and quickly frozen in liquid nitrogen. No patients were treated before undergoing surgical resection. The adjacent nonmalignant lung tissues which were away from the cancer tissues at least 5 cm, did not contain cancer cells but usually appeared inflammatory response and fibrosis. Pathological diagnosis was based on light microscopy according to the World Health Organization classification [12]. Tumors were staged according to TNM criteria published by the International Union Against Cancer in 1997 [13]. Tumor regions selected for RNA and protein isolation contained a tumor cellularity greater than 60%. The use of all of the human tissue samples and the experimental procedures for this study were reviewed and approved by the Tumor Hospital of Yunnan Province and Kunming Institute of Zoology. All researches were carried out according to the Helsinki Declaration.

### Cell culture

Six lung cancer cell lines and an immortalized human bronchial epithelial cell line (BEAS-2B) were used. A549 (AC), 801-D (large cell carcinoma), NCI-H446 (SCLC), and BEAS-2B were obtained from Cell Bank of Kunming Institute of Zoology, Chinese Academy of Sciences (CAS, Kunming, China); SPCA-1 (AC) was purchased from the Cell Bank of Type Culture Collection, CAS (Shanghai, China); EPLC-32M1 (SCC) and GLC-82 (AC) were obtained from German Cancer Research Center (Heidelberg, Germany) and Chinese National Cancer Institute, Chinese Academy of Medical Sciences (Beijing, China), respectively. The BEAS-2B cell line was fed with DMEM, while the other cell lines were cultured with RPMI 1640 containing 10% fetal bovine serum (FBS) and maintained in a humidified incubator with 5% CO_2_ at 37°C.

### Construction of the subtractive cDNA library

To construct the SSH library, we used a tumor tissue and its adjacent nonmalignant lung tissue derived from one patient with well-differentiated lung adenocarcinoma. The tumor tissue contained a tumor cellularity greater than 80%. The adjacent nonmalignant lung tissue was away from the cancer tissues at least 10 cm and appeared completely normal histological structure. Total RNA was isolated using TRIzol (Invitrogen Corp., Carlsbad, CA, USA). Poly(A)^+^ RNA was isolated using the PolyATtract® mRNA isolation systems (Promega Corp., Madison, WI, USA). The cDNA synthesis and subtraction were performed using the PCR-select™ cDNA subtraction kit (Clontech, Palo Alto, CA, USA). Using the cDNA of lung adenocarcinoma tissue as tester and its adjacent nonmalignant lung tissue as driver, we generated the FSL which represented up-regulated transcripts. Using the cDNA of lung adenocarcinoma tissue as driver and its adjacent nonmalignant lung tissue as tester, the RSL which represented down-regulated transcripts, was constructed. The subtracted cDNA fragments obtained in each experiment were cloned into the pGEM-T Easy vector (Promega Corp.) and used to transform E.coli strain DH5α cells. All the transformants were isolated from white colonies on X-gal/isopropyl-beta-D-thio-galatopyranoside agar plates.

### Quantitative real-time polymerase chain reaction (q-RT-PCR)

Total RNA was isolated from lung cancer tissues and adjacent nonmalignant tissues using TRIzol (Invitrogen Corp.). RNA was used as a template to synthesize the first strand cDNA with M-MLV reverse transcriptase (Promega Corp.). The q-RT-PCR reactions were performed on an ABI stepone™ real-time RT-PCR system using the SYBR Green dye method. The q-RT-PCR reactions were performed in 25 μl volumes that included 0.5 μl of SYBR green, 8 ng of cDNA template and 1.0 μl each of the forward and reverse primers (10 μM) (see Additional file 1). The PCR was done under the conditions at 94°C for 30 seconds, then 40 cycles of amplification at 94°C for 30 seconds, 60°C for 30 seconds. Each gene was normalized to the internal β-actin levels. Each sample was run in triplicate to ensure quantitative accuracy, and the threshold cycle numbers (Ct) were averaged. The results were reported as tumor tissues: normal tissues (T:N) ratios and calculated using the 2^−ΔΔCt^ method [14].

### Western blot

Total protein was extracted from cultured cells and tissues, separated by electrophoresis, and then transferred into PVDF membrane according to the routine protocol. The blotted membrane was incubated with the rabbit anti-ERGIC3 polyclonal antibody (Sigma-Aldrich, St. Louis, MO, USA) followed by horseradish peroxidase-conjugated anti-rabbit secondary antibody (AbMART, Shanghai, China), and then proteins were detected with chemiluminescence reagents (Thermo Fisher Scientific Inc., Waltham, MA, USA). The membrane was reprobed with an antibody against β-actin (Abcam, Cambridge, UK) as a control for equivalent protein loading.

### Immunohistochemical staining

Immunohistochemical staining was done on 4-μm-thick paraffin sections cut from the formalin fixed tissues. Heat-induced epitope retrieval was performed in Tris-EDTA Buffer (10 mM Tris Base, 1 mM EDTA Solution, 0.05% Tween 20, pH 9.0). The sections were then incubated in 3% H_2_O_2_ for 10 minutes to eliminate endogenous peroxidase activity. The primary rabbit anti-ERGIC3 polyclonal antibody (Abcam) and horseradish peroxidase-conjugated anti-rabbit secondary antibody were used. Color development was accomplished with 3,3'-Diaminobenzidin. The nuclei were then counterstained with hematoxylin. Only manifest cytoplasmic staining was defined as a positive reaction. Negative controls were incubated with normal rabbit serum instead of the polyclonal antibody.

### Immunofluorescence staining and confocal microscopy

Immunofluorescence analysis was done on the cultured cells. Briefly, the cells grown on coverslips were fixed in −20°C acetone for 10 minutes and then incubated with the rabbit anti-ERGIC3 polyclonal antibody (Abcam), and anti-MUC1 mouse monoclonal antibody (mAb) A76-A/C7 (Glycotope, Berlin, Germany), anti-ST mAb [15], anti-calreticulin mAb (Abcam), and anti-58K Golgi protein mAb (Abcam) at 4°C overnight. Following the washes, the cells were incubated with FITC-coupled anti-rabbit antibody (BD sciences, Franklin Lakes, NJ, USA) or with Cy3-coupled anti-mouse antibody (Millipore, Billerica, MA, USA) for 1 hour. Subsequently, the nuclei were finally counterstained with diamidinophenylindole. The slides were analyzed under confocal laser scanning microscopy.

### Construction of the expression vector and the cell transfection

To generate the plasmid expressing ERGIC3, we cloned the open reading frame of *ERGIC3* and used the following specific primers (upper case, restriction enzyme sequences): forward, 5'-GGTGGTGAATTC*atgaggcgctggggaagct*-3'; reverse,5'-GGTGGTGGATCCaccgaggagggtgactacgttgtctt-3'. After running PCR, the product was cut by EcoR I and BamH I. The purified DNA was ligated into the pLXSN expression vector (Clontech Corp.). The empty vector served as a control. The vectors were transfected into BEAS-2B cell by lipofectamine LTX and PLUS (Invitrogen Corp.) according to the instructions. The over-expression of ERGIC3 was validated by q-RT-PCR and western blot.

### Gene silencing by RNA interference

For gene silencing of ERGIC3, pGPU6/GFP/Neo-ERGIC3-shRNA and its control vector, pGPU6/GFP/Neo-NC-shRNA, were used (Jima Corp., Shanghai, China). Briefly, the oligonucleotide GGAGGACTATCCAGGCATTGT was designed to interfere specifically with ERGIC3 gene expression. As a negative control, we used the oligonucleotide GTTCTCCGAACGTGTCACGT, which has no significant match in a BLASTn search (human NCBI nr database). The vectors were transfected into GLC-82 cells by lipofectamine LTX and PLUS. The gene silencing of ERGIC3 was validated by q-RT-PCR and western blot.

### Cell proliferation assay

Cell proliferation analysis was based on the capacity of mitochondrial enzymes to transform MTT to MTT formazan. More succinctly, after being transfected for 48 hours, GLC-82 and BEAS-2B were collected and transferred into 96-well plates (4*10^3^ cells/well), then all cells were treated with MTT (5 mg/ml) every 24 hours. 10 μl of MTT was added to each well and cells were incubated at 37°C for 2 hours. Then, the culture medium with dye was removed and dimethylsulfoxide was added at 100 μl per well for formazan solubilization. The absorbance of converted dye was measured at a wavelength of 490 nm using a 96-well microplate reader (model 680, Bio-Rad Laboratories).

### Cell migration assay

The transfected GLC-82 and BEAS-2B cells were used for the cell migration assay. The transwell migration assay was conducted in 24 well plates with membrane inserts (8 mm pore size; Millipore). The cells were then seeded in the upper chamber of transwells (10^5^ cells/well) without FBS. The lower chambers were loaded with 500 μl of medium with 10% FBS. After incubation for 24 hours, filters were washed and the cells on the upper surface were gently removed with a cotton swab. The cells were fixed with 95% ethanol, and were stained by Giemsa I (Jiancheng Corp. Nanjing, China) and Giemsa II. The cells were then counted under microscope. The experiments were repeated three times.

### Statistical analysis

Measurement data (levels of mRNA and protein, the data of cellular migration and proliferation) and enumeration data (the data of immunohistochemical staining) were respectively analyzed using the paired *t*-test and the Fisher’s exact test (two-tailed). All of the values were evaluated using IBM SPSS 19 (SPSS inc., Chicago, Illinois). Differences were considered significant if *P* < 0.05.

## Results

### Generation of SSH cDNA libraries

Using SSH, two cDNA libraries were constructed with the primary lung AC tissue derived from a single patient. From the FSL library, 485 subtractive cDNA clones were obtained, representing genes up-regulated in tumor tissues. Meanwhile, from the RSL library, 172 subtractive cDNA clones were obtained, representing genes down-regulated in tumor tissues. Using the BLAST algorithm at NCBI RefSeq, GenBank, and dbEST, we detected 177 genes and 44 unknown expressed sequence tags (ESTs) in the FSL as well as 59 genes and 10 unknown ESTs in the RSL. Further bioinformatic analysis demonstrated the major functions of these genes were related molecular transport, cellular signaling and interaction, cellular polarization, cell cycle, cell apoptosis, cellular growth and proliferation, cellular movement, DNA replication, recombination and repair (see Additional file 2 and 3).

Eight earlier constructed SSH libraries of lung cancer (five FSLs and three RSLs) were available for us to obtain gene expression profiles [6-10]. From the previous five FSLs, 152 genes from our FSL were not reported. Similarly, 54 genes in our RSL were not present in the previous three RSLs. In the six aggregated FSLs from our study and earlier publications, 42 genes appeared twice, while four genes (*EEF1A1*, *FTH1*, *GSTP1*, *STAT1*) appeared three times (see Additional file 4). Additionally, in the four RSLs from our study and the three previous, only six genes (*ANXA8*, *CAV1*, *CEBPD*, *GPX3*, *TPM3*, *NACA*) appeared twice. Among those six, *CAV1*, *CEBPD*, *GPX3*, *TPM3* and *NACA* were present in our RSL (see Additional file 5).

### mRNA expression of a part of differentially expressed genes in lung cancer tissues

As a first stage analysis, we selected 16 genes among the differentially expressed genes from our FSL and RSL libraries for further study based on two criteria: 1) The 10 genes were previously reported in lung cancer while six were not reported; 2) These genes belong to importantly functional genes. We examined mRNA expression of 16 genes selected from the SSH libraries using q-RT-PCR. Among the 16 genes, two were selected from the RSL, one from both the RSL and FSL, and 13 from the FSL. The percentage of the altered expression in the tumor tissues compared to their adjacent nonmalignant lung tissues is shown in Table 1. The mRNA levels of the 16 genes in the lung cancer tissues were compared with those in the adjacent nonmalignant lung tissues using the paired *t*-test. The two genes selected from the RSL were significantly down-regulated in the tumor tissues as compared with the adjacent nonmalignant lung tissues (*GPX3*, *P* = 0.000 and *TIMP3*, *P* = 0.000), and six genes selected from the FSL were significantly up-regulated in the lung cancer tissues (*DDR1*, *P* = 0.009; *HSP90B1*, *P* = 0.000; *SDC1*, *P* = 0.007; *RPSA*, *P* = 0.013; *ERGIC3*, *P* = 0.000; and *LPCAT1 P* = 0.036). However, seven genes selected from FSL (*FOXA2*, *C4BPA*, *SCGB3A1*, *DDX58*, *CCNDBP, TMSB4X*, and *CXCL17*), and one gene selected from both FSL as well as RSL (*CD9*), were up-regulated in the lung cancer tissues, but not significantly (*P* > 0.05). The tendency of the seven genes expressions was consistent with that of the FSL which represented genes up-regulated in cancer tissues. We noted that *CD9*, present in both the FSL and RSL, was increased in 41.2% of lung cancer cases. Perhaps the presence of *CD9* in the RSL was a false signal. Over-expression of two genes (*ERGIC3* and *LPCAT1*) had not been previously linked to lung cancer. We opted to focus on *ERGIC3* in this study.

**Table 1:** Altered expression of the 16 genes in lung cancer tissues compared with their adjacent nonmalignant lung tissues using quantitative RT-PCR (q-RT-PCR)

**Gene**	**Library**	**Percentage**	**Main Functions**	**References**
GPX3	RSL	↓ 85% (29/34)^#^	Detoxification of hydrogen peroxide. Suppresses prostate cancer growth and metastasis.	Brigelius *et al.* (2012) Yu *et al.* (2007)
TIMP3	RSL	↓ 85% (29/34)	Degradation of the extracellular matrix. A mediator for checking inflammation. Cell invasion, proliferation, and death.	Gomez *et al.* (1997) Shao *et al.* (2012) Baker *et al.* (1998)
CD9	RSL & FSL	↑ 41% (14/34)	Functions in many cellular processes including differentiation, adhesion, and signal transduction. Suppression of cancer cell motility and metastasis.	Chen *et al.* (2011) Yamazaki *et al.* (2011)
DDR1	FSL	↑ 74% (25/34)	Communication of cells with their microenvironment. Regulation of cell growth, differentiation and metabolism.	Valencia *et al.* (2012) Ruiz *et al.* (2011) Kim *et al.* (2011)
HSP90B1	FSL	↑ 82% (28/34)	Molecular chaperones with roles in stabilizing and folding other proteins. Association with a variety of pathogenic states.	Sanz-Pamplona *et al.* (2011) Eletto *et al.* (2010) Calderwood *et al.* (2007)
FOXA2	FSL	↑ 29% (10/34)	Transcriptional activators for liver-specific genes. Maintenance of glucose and lipid homeostasis. Suppressor of tumor metastasis by inhibition of EMT.	Rausa *et al.* (2004) Wolfrum *et al.* (2004) Tang *et al.* (2011)
SDC1	FSL	↑ 82% (28/34)	Regulation of cell proliferation, cell migration and cell-matrix interactions.	Schmedt *et al.* (2012) Zong *et al.* (2011)
C4BPA	FSL	↑ 35% (12/34)	Activation of the complement cascade.	Arenzana *et al.* (1995) Fraczek *et al.* (2010)
SCGB3A1	FSL	↑ 56% (19/34)	Inhibition of cell growth and invasion.	Haakensen *et al.* (2011) Tomita *et al.* (2009)
RPSA	FSL	↑ 65% (22/34)	Implication of biological processes including cell adhesion, differentiation, migration, signaling, neurite outgrowth and metastasis.	Qiao *et al.* (2009) Zidane *et al.* (2012)
ERGIC3	FSL	↑ 76% (47/62)	Regulation of cell growth and endoplasmic reticulum stress-induced cell death. Protein transport through the early secretory pathway.	Zhang *et al.* (2012) Nishikawa *et al.* (2007) Otte *et al.* (2002)
DDX58	FSL	↑ 47% (29/62)	Regulation of immune response.	Liu *et al.* (2011) Aida *et al.* (2011)
CCNDBP1	FSL	↑ 47% (29/62)	Inhibition of cell cycle. Suppression of tumorigenesis.	Lee *et al.* (2010) Ma *et al.* (2007)
TMSB4X	FSL	↑ 37% (23/62)	Regulation of actin polymerization. Implication of cell proliferation, migration, and differentiation	Moon *et al.* (2010) Selmi *et al.*(2012)
CXCL17	FSL	↑ 45% (28/62)	Anti-inflammatory factor. Promotion of angiogenesis.	Lee *et al.* (2012) Matsui *et al.* (2012)
LPCAT1	FSL	↑ 47% (29/62)	Catalyzing the conversion of LPC to phosphatidylcholine (PC) in the remodeling pathway of PC biosynthesis. Promotion of cell growth.	Nakanishi *et al.* (2006) Soupene *et al.* (2012) Mansilla *et al.* (2009)

↓: Down-regulation of the genes in lung cancer tissues compared with their adjacent nonmalignant lung tissues; ↑: Up-regulation of the genes in lung cancer tissues compared with their adjacent nonmalignant lung tissues; #: Number of cases applicable/total number of cases examined.

### Expression of ERGIC3 mRNA in cultured cells

Similar to the results we found in lung cancer tissues, in the lung cancer cell lines, the mRNA levels of *ERGIC3* showed up to 44.9- (in SPCA-1), 61.4- (in EPLC-32M1), 60.8- (in GLC-82), 22.1- (in NCI-H446), 16.0- (in A549), 32.1- (in 801D) fold increase, compared to the immortalized normal bronchial epithelial cells, BEAS-2B.

### Expression of ERGIC3 protein in cultured cells and in lung cancer tissues by western blot

**Figure 1:**
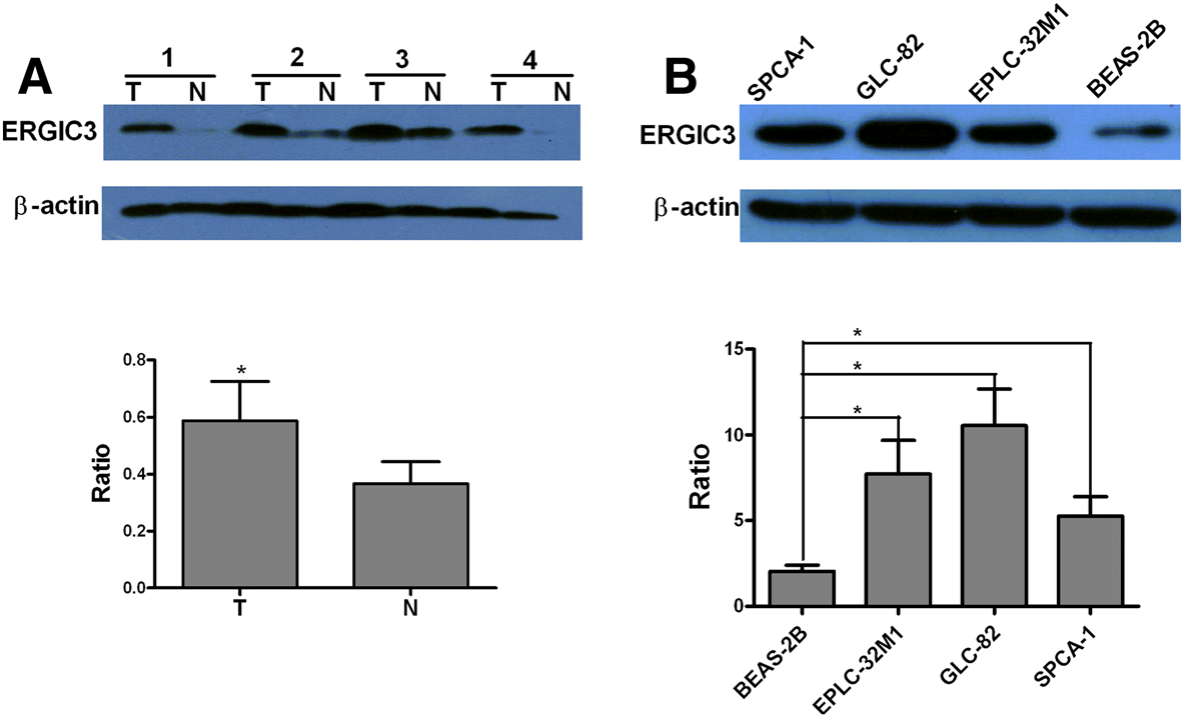
**Semi-quantitative analysis of the ERGIC3 protein by western blot. (A)** The averages of protein levels in the 15 tumor tissues (T) and their adjacent nonmalignant tissues (N). **(B)** The averages of protein levels in three separate experiments on SPCA-1, GLC-82, EPLC-32M1 and BEAS-2B. The Y-axis shows the ratio of the ERGIC3 grey value divided by the beta-actin grey value. The significance was evaluated by a paired *t*-test (two tailed). *: *P* < 0.05.

Expression of the ERGIC3 protein was analyzed using western blot. Expression of ERGIC3 was increased in 67% (10/15) of the tumor cases (Figure 1A). Similarly, expression of ERGIC3 protein was increased in all three lung cancer cell lines by comparison with the BEAS-2B (Figure 1B).

### Subcellular localization of ERGIC3 protein in cultured cells

**Figure 2:**
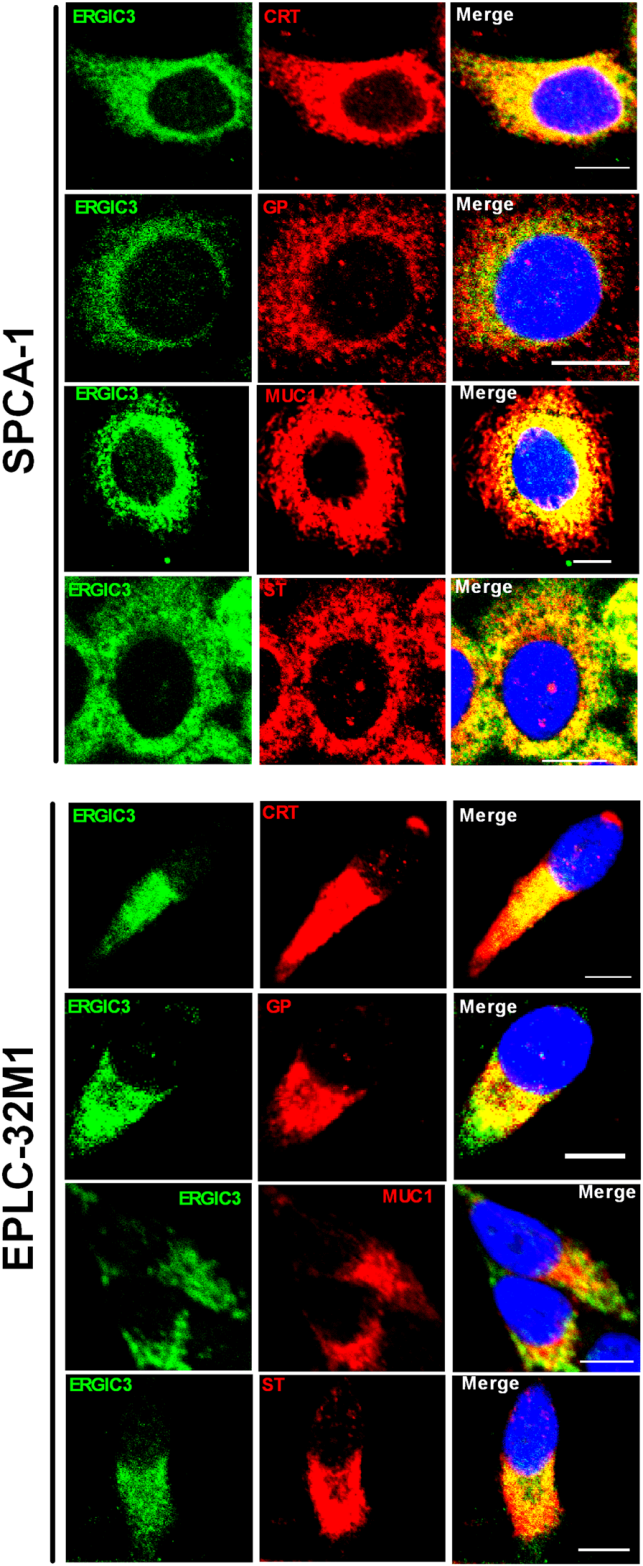
**Subcellular colocalization of ERGIC3 in cultured cells.** ERGIC3 [green] with calreticulin (CRT), Golgi protein (GP), MUC1, and β-galactoside α2,6 sialyltransferase (ST) [red] in lung cancer cell lines, SPCA-1 and EPLC-32M1. Nuclei were stained by diamidinophenylindole [blue]. Scale bar: 10 μm.

In cultured cells, the subcellular localization of ERGIC3 was examined by immunofluorescence double-staining using markers of the Golgi apparatus and endoplasmic reticulum (ER). ERGIC3 was mainly located at the Golgi apparatus and ER in the lung cancer cell lines. Interestingly, ERGIC3 was distributed at the side of nucleus in EPLC-32M1, 801D, and NCI-H446 cells, but uniformly present around nucleus in SPCA-1, GLC-82 and A549 cells (Figure 2).

We also found that ERGIC3 was co-localized with the epithelia mucin MUC1 and β-Galactoside α2,6 Sialyltransferase (ST), which were principally located at the ER and Golgi apparatus in the cultured cells (Figure 2).

### Expression and localization of the ERGIC3 protein in lung cancer tissues by immunohistochemical staining

**Figure 3:**
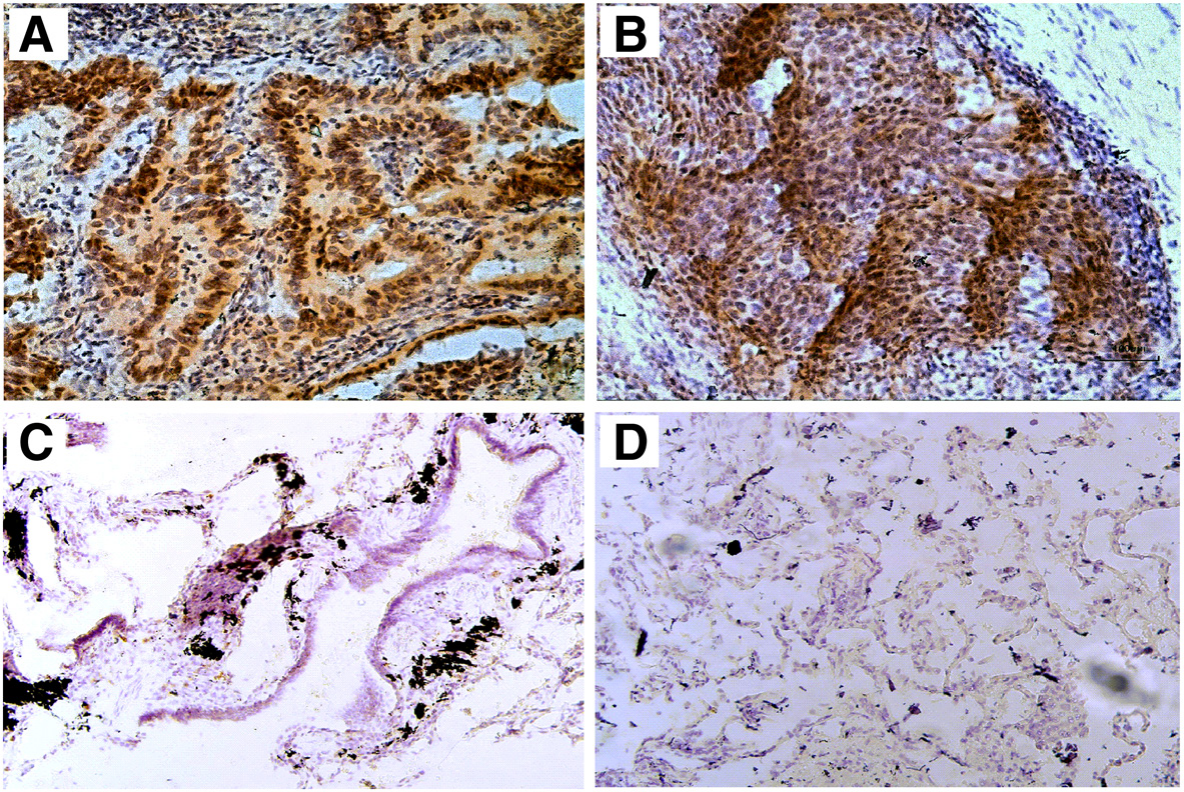
**Immunohistochemical staining of ERGIC3 in the lung cancer tissues and normal lung tissues.** ERGIC3 was strongly stained in the cytoplasm of tumor cells on lung adenocarcinomas **(A)** and squamous cell carcinomas **(B)**, but it was negative in the ciliated epithelium **(C)** and normal respiratory epithelium **(D)**. Original magnification, ×200 **(A.B)**, ×100 **(C.D)**.

Expression and localization of the ERGIC3 protein was further investigated by immunohistochemistry in 35 cases of NSCLC. ERGIC3 was diffusely distributed in the cytoplasm of tumor cells but not expressed in normal bronchial epithelial cells and alveolar cells (Figure 3). The antibody of ERGIC3 used in the study was the polyclonal anti-ERGIC3 serum, this may be the reason that the non-specific staining appeared in nucleus.

ERGIC3 was positive in 89% of (31/35) NSCLCs, and strongly stained in 63% (22/35). Interestingly, the positive rate of AC (100%, 22/22) was higher than that of SCC (69%, 9/13). In poorly differentiated NSCLCs, 36% (4/11) cases were not stained. We noticed that all of negative specimens were poorly differentiated tumor cells, and the staining was decreased to the largest extent and even disappeared in poorly differentiated NSCLCs, compared with well and moderately NSCLCs (*P* <0.05). No correlations were observed between expression of ERGIC3 and the gender as well as smoking histories of the patients and TNM stage (Table 2).

**Table 2:** Immunohistochemical staining of ERGIC3 in lung cancer tissues

**Characteristic**	**ERGIC3**^**−**^	**ERGIC3**^**+**^	**P-value**
Gender			0.279
Male	3/17 (18%)^#^	14/17 (82%)	
Female	1/18 (6%)	17/18 (94%)	
Age, y			/
Median	55.9	53.5	
Range	36-76	38-71	
Smoking			0.323
No	1/17 (6%)	16/17 (94%)	
Yes	3/18 (17%)	15/18 (83%)	
Histological type			0.014*
AC	0	22/22 (100%)	
SCC	4/13 (31%)	9/13 (69%)	
TNM stage			0.419
I + II	3/20 (15%)	17/20 (85%)	
III + IV	1/15 (7%)	14/15 (93%)	
Differentiation			0.006*
Poor	4/11 (36%)	7/11 (64%)	
Well or Moderate	0	24/24 (100%)	

Statistical significance was determined by the Fisher’s exact test of chi-square test (two-tailed); #: Number of cases applicable/total number of cases examined (with percent in parenthesis); *: Statistically significant, *P* < 0.05.

Abbreviations: SCC: squamous cell carcinoma; AC: adenocarcinoma; y: year.

### Effects of ERGIC3 expression on the proliferation of the epithelial cells

**Figure 4:**
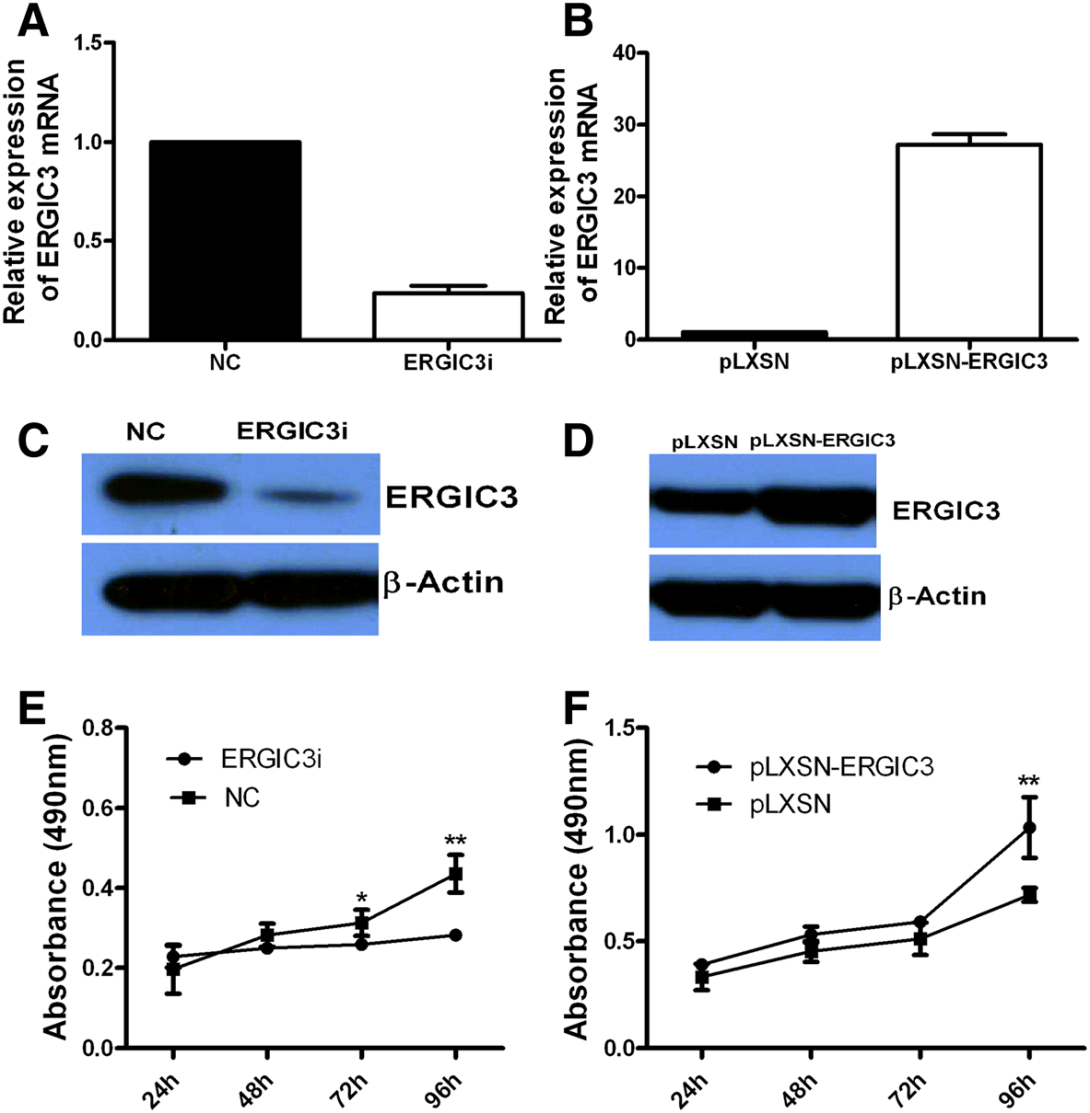
**Expression of ERGIC3 and cellular proliferation.** The levels of *ERGIC3* mRNA were evaluated by q-RT-PCR in GLC-82 cells after ERGIC3 gene silencing **(A)** and in BEAS-2B cells after ERGIC3 over-expression **(B)**. And the levels of ERGIC3 protein were evaluated by western blot in GLC-82 cells after ERGIC3 gene silencing **(C)** and in BEAS-2B cells after ERGIC3 over-expression **(D)**. Reduced expression of ERGIC3 could slow the proliferation rates of GLC-82 cells **(E)**, but increased expression of ERGIC3 could enhance the proliferation rates of BEAS-2B cells **(F)**. Differences in cell proliferation rates were analyzed by a paired *t*-test (two tailed). NC: negative control; ERGIC3i: ERGIC3 gene silencing; pLXSN: treated with the pLXSN vector; pLXSN-ERGIC3: treated with the pLXSN-ERGIC3 vector. *: *P* < 0.05; **: *P* < 0.01.

In order to study whether altered expression of ERGIC3 affects the cell proliferation, GLC-82 (with the high level of endogenous ERGIC3) and BEAS-2B cells (with the low level of endogenous ERGIC3) were used in the study. In GLC-82 cells, compared with the cells transfected with the control vector, the expression of ERGIC3 was reduced by RNA interference. While the expression of ERGIC3 in BEAS-2B cells was increased after the transfection with the expression vector. The altered expression of ERGIC3 was indicated by q-RT-PCR (Figure 4A,B) and western blotting (Figure 4C,D). We found the reduced expression of ERGIC3 in GLC-82 significantly reduced the rate of cell proliferation (Figure 4E), and the over-expression of ERGIC3 in BEAS-2B cells significantly increased the rate of cell proliferation (Figure 4F).

### Effects of ERGIC3 expression on the cellular migration

**Figure 5:**
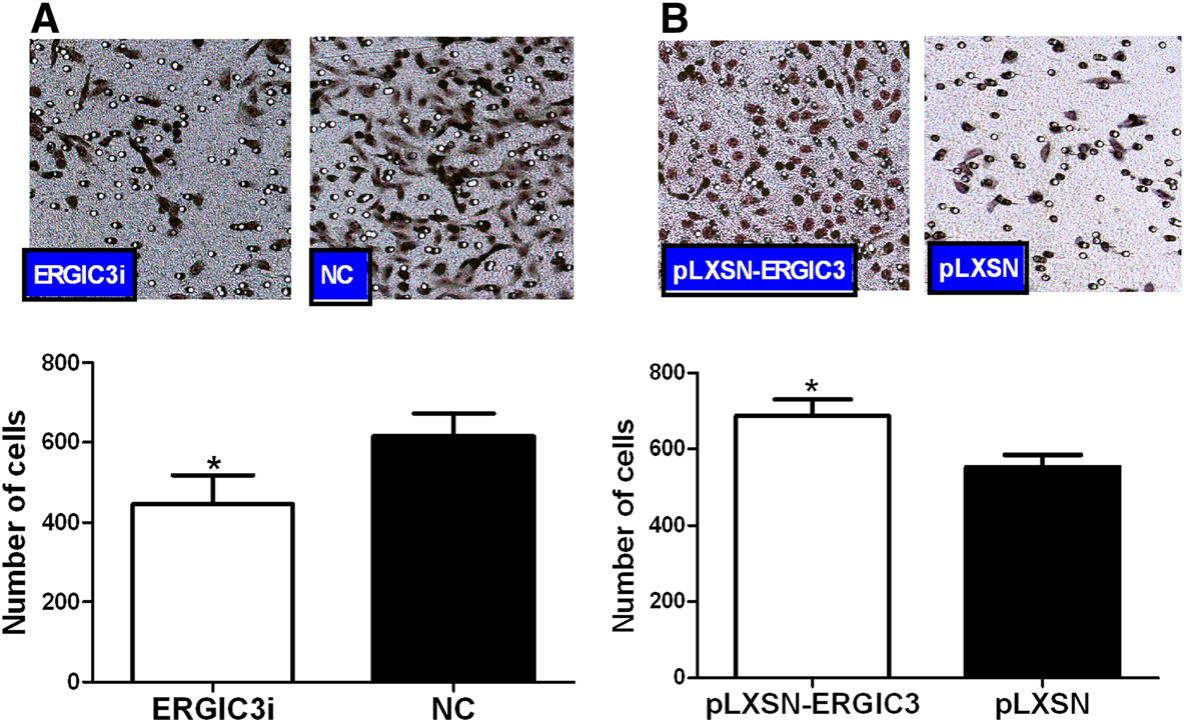
**Expression of ERGIC3 and cellular migration.** Reduced expression of ERGIC3 could demote the migration of GLC-82 cells **(A)**, but increased expression of ERGIC3 could promote the migration of BEAS-2B cells **(B)**. Differences in numbers of migration cells were analyzed by a paired *t*-test (two tailed). NC: negative control; ERGIC3i: ERGIC3 gene silencing; pLXSN: treated with the pLXSN vector; pLXSN-ERGIC3: treated with the pLXSN-ERGIC3 vector. *: *P* < 0.05

We also analyzed the impact of ERGIC3 on cellular migration. The expression of ERGIC3 in GLC-82 and BEAS-2B cells was successfully altered through gene silencing and over-expression, as described earlier. The treated cells were used for the transwell migration assay based on Boyden Chamber. We found that the lower-expression of ERGIC3 could inhibit the cellular migration in GLC-82 (Figure 5A), and the over-expression of ERGIC3 could promote the cellular migration in BEAS-2B (Figure 5B).

## Discussion

Differentially expressed genes are the fundamental driver of both the diversity and complexity of tumor phenotypes. Generating subtracted cDNA libraries is the first step in identifying genes differentially expressed in cancer cells. This construction is made easier by the simple and highly effective suppression subtractive hybridization (SSH) technique. SSH has been successfully applied to a wide variety of malignant diseases for the generation of cDNA libraries [6-10].

In this study, we constructed two libraries of differentially expressed genes using the lung AC tissue and its adjacent nonmalignant lung tissue from a single patient. We compared and analyzed the results from all the lung cancer SSH libraries, including our own. Surprisingly, most of the differentially expressed genes were unequal in these libraries. This could be explained that diverse cancer tissues and cell lines lead to different gene expression profiles. The genetic abnormality of cancer appears a great diversity. Accordingly, using various samples to generate new cDNA libraries in order to find novel differentially expressed genes in lung cancer can play a significant role in advancing research into lung cancer. In this regard, our SSH libraries add useful data that can further be used in the discovery of lung cancer-associated genes. Indeed, the vast majority of the differentially expressed genes of our libraries were not known to be abnormally expressed in lung cancer.

Bioinformatics analysis was subsequently performed to interpret the differentially expressed genes of our FSL and RSL libraries. The functions of these genes were involved in several aspects: molecular transport; cellular signaling and interaction; cellular polarization; cell cycle; DNA replication, recombination, and repair; cell apoptosis; cellular growth and proliferation; cellular movement. Here we would like to emphasize several interesting facts observed during this study. 1) 12.4% (22/177) of our FSL was ribosomal protein genes but these proteins only made up 1.7% (1/59) of the genes found in our RSL. The large amount of ribosomal proteins up-regulated in the lung AC tissues from this data seems to indicate that thriving cancer cells need plenty of ribosomal proteins. 2) Cancer is a multi-genetic disease. Individual tumors accumulate an average of 90 mutant genes [16]. In our study, the results based on the two libraries of differentially expressed genes demonstrated that in a given tumor more than 200 genes simultaneously exhibited abnormal expression and the number of the up-regulated genes was more than that of the down-regulated genes, indicating that even in a tumor the genetic abnormality is greatly extensive and complex. Although the case which was used to construct the libraries is very limited, the study would also have provided clues to the trend of gene expression alterations.

A large fraction of the differentially expressed genes found in cancer were likely to be “passenger” genes that are not to be integral to neoplasia. However, screening of “non-passenger” genes (functionally important alterations) among the differentially expressed genes and studying their functions are helpful for the elucidation of tumorigenesis mechanisms and the discovery of new diagnostic and therapeutic targets. As a first stage analysis, 16 genes were selected among the differentially expressed genes from our FSL and RSL libraries for further study. Through q-RT-PCR analysis, we found six genes (*DDR1*, *HSP90B1*, *SDC1*, *RPSA*, *ERGIC3*, and *LPCAT1*) significantly up-regulated and two genes (*GPX3* and *TIMP3*) significantly down-regulated in the lung cancer tissues. Among the eight genes significantly up- or down-regulated in the lung cancers in this study, the six genes have been noted in previous studies for the connection between their expression and lung cancer: up-regulated *DDR1*[17], *HSP90B1*[18], *SDC1*[19], and *RPSA*[20], as well as down-regulated *GPX3*[21] and *TIMP3*[22]. Importantly, this means that for the first time, over-expression of *ERGIC3* and *LPCAT1* have been linked to lung cancer. *LPCAT1* may contribute to total choline metabolite accumulation via phosphatidylcholine remodeling, thereby altering the colorectal cancer lipid profile, a characteristic of malignancy [23], but this was investigated in a separate work as the current study focuses on ERGIC3.

ERGIC3 (previously labeled Erv46, ERp43) is an integral membrane protein that cycles between the ER and Golgi [24,25]. ERGIC3 has large domains in the ER lumen, and its short N- and C-terminal tail sequences expose to the cytosol and the two transmembrane segments each, which would be available for protein-protein interactions in the ER lumen or membrane [26]. Through the systematic and serial screening, we found that the ERGIC3 mRNA and protein were highly over-expressed in lung cancer cells. In the cultured cells, ERGIC3 was mainly located at the Golgi apparatus and ER, in agreement with previous descriptions [11]. We observed that the distribution of ERGIC3 was associated with the cellular shape. In the round cells, ERGIC3 was located around the nucleus, but it was at the side of the nucleus in the fusiform cells. This phenomenon indicates that the localization of ERGIC3 is identical to the distribution of the Golgi apparatus and ER. ERGIC3 may be a well marker of the Golgi apparatus and ER in cancerous cells.

We also found that ERGIC3 was closely co-localized with MUC1 and ST. MUC1 is a high molecular weight transmembrane glycoprotein. The protein backbone of MUC1 is synthesized at the ER and glycosylated at the Golgi apparatus. ST is a key enzyme that sialylates lactosaminyl termini of complex type oligosaccharides in an α2,6 linkage. ST is located predominantly in the trans-Golgi network, and could be secreted by some cells [27]. ERGIC3 is involved in the protein transport between the ER and the Golgi apparatus. We guess that ERGIC3 could participate in the transport of MUC1 and ST from the ER to the Golgi apparatus. Further studies will hopefully address this issue.

In our study, ERGIC3 was found positive in 89% specimens of lung cancer by immunohistochemical staining. In contrast, ERGIC3 was not expressed in normal bronchial epithelial cells and alveolar cells. These results from our pilot study suggested that ERGIC3 may be a potential biomarker for lung cancer. However, more studies must be performed to permit final conclusions. Our laboratory has already begun the relevant research.

We further investigated pathophysiological functions of the altered expression of ERGIC3 in lung cancer cells. In our study, over-expressed ERGIC3 promoted the cellular proliferation. A recent study demonstrated that ERGIC3 played important roles in cell growth and ER stress-induced apoptosis [28]. Additionally, we also found that up-regulation of ERGIC3 facilitated cellular migration. The cellular proliferation and migration are essential events during carcinogenesis and cancerous invasion. ERGIC3 may play an active role in the development and progression of lung cancer. At present, the full mechanisms by which ERGIC3 promotes cellular proliferation and migration are not understood. Previous studies demonstrated that Erv41p-Erv46p complex interacts with glucosidase II and modulates glucosidase I activity, as well as cells lacking a cycling Erv41p-Erv46p complex display a mild glycoprotein processing defect [26], and the mutation of ERGIC3 could reduce the transport between the ER and the Golgi apparatus [29]. It is then tempting to speculate that abnormally expressed ERGIC3 could affect the cellular proliferation and migration through the disruption of glucosidase activity and protein intracellular transport.

## Conclusions

We used SSH to generate two cDNA libraries (FSL and RSL) of differentially expressed genes. The 177 up-regulated and 59 down-regulated genes in lung cancer were obtained. The vast majority of these genes were linked to lung cancer for the first time. In the first stage of the screening for 16 genes, two novel lung cancer-related genes (*ERGIC3* and *LPCAT1*) were found. ERGIC3 was strongly expressed in lung cancers, and that ERGIC3 could promote the cellular proliferation and migration. These findings suggest that *ERGIC3* may play an active role in the development and progression of lung cancer. Consequently, our two libraries of differentially expressed genes may provide the basis for new insights or clues for finding novel lung cancer-related genes. Hopefully, several serious studies will be made on the data we collected in these two libraries.

## Abbreviations

ERGIC3: Endoplasmic reticulum-Golgi intermediate compartment protein 3; SCLC: Small-cell lung cancer; NSCLC: Non-small cell lung cancer; AC: Adenocarcinoma; SCC: Squamous cell carcinoma; SSH: Suppression subtractive hybridization; FSL: Forward-subtracted library; RSL: Reverse-subtracted library; EST: Expressed sequence tag; q-RT-PCR: Quantitative real-time polymerase chain reaction; mAb: Mouse monoclonal antibody; FBS: Fetal bovine serum; ER: Endoplasmic reticulum; ST: β-Galactoside α2,6 Sialyltransferase.

## Competing interests

The authors declare no conflict of interest.

## Authors’ contributions

MW carried out the molecular biology studies, participated in the design of study and drafted the manuscript. TT carried out the immunoassays, participated in the molecular biology studies. YH provided the clinical materials, participated in the analysis of the data. YC conceived of the study, participated in its design and coordination, and helped to draft the manuscript. All authors read and approved the final manuscript.

## Pre-publication history

The pre-publication history for this paper can be accessed here:

http://www.biomedcentral.com/1471-2407/13/44/prepub

## Supplementary Material

Additional file 1 Primer sequences for real-time RT–PCR. Click here for file

Additional file 2 Representative differentially expressed genes with identified chromosome locations in the forward-subtracted library of primary lung adenocarcinoma. Click here for file

Additional file 3 Representative differentially expressed genes with identified chromosome locations in the reverse-subtracted library of lung adenocarcinoma. Click here for file

Additional file 4 The genes appeared twice or three times in the different forward- subtracted libraries of lung cancer by suppression subtractive hybridization. Click here for file

Additional file 5 The genes appeared twice in the different reverse-subtracted libraries of lung cancer by suppression subtractive hybridization. Click here for file
