# Supplementary material for: Suppression subtractive hybridization identified differentially expressed genes in lung adenocarcinoma: ERGIC3 as a novel lung cancer-related gene
Source: BMC Cancer. 2013 Feb 1;13:44. doi: 10.1186/1471-2407-13-44 (PMC3567939; doi:10.1186/1471-2407-13-44)
Supplement: Additional file 1 — Primer sequences for real-time RT–PCR. [file 1471-2407-13-44-S1.doc]

**Additional file 1. Primer sequences for real-time RT–PCR**

| Gene | Primer sequence |
| --- | --- |
| TIMP3 | F:5’-CAACTCCGACATCGTGATCCG-3’ |
|  | R:5’-GAAGCCTCGGTACATCTTCATC-3’ |
| GPX3 | F:5’-TTGATGGGGAGGAGTACATCC-3’ |
|  | R:5’-AGACCGAATGGTGCAAGCTC-3’ |
| RPSA | F:5’-TCCTGCAAATGAAGGAGGAGG-3’ |
|  | R:5’-CCATCTGGAAGTCAAGATTGGTG-3’ |
| DDR1 | F:5’-AGATGCTGACATGAAGGGACA-3’ |
|  | R:5’-GGCAGTGGAATCTGACCAGG-3’ |
| SDC1 | F:5’-TGAAACCTCGGGGGAGAATAC-3’ |
|  | R:5’-GGTACAGCATGAAACCCACC-3’ |
| HSP90B1 | F:5’-CTGGGACTGGGAACTTATGAATG-3’ |
|  | R:5’-TCCATATTCGTCAAACAGACCAC-3’ |
| ERGIC3 | F:5’-GGAGAGGTACTGAGGACAAATCA-3’ |
|  | R:5’-AGCTCATAGAGGACGAAGACTC-3’ |
| CD9 | F: 5’-GGATATTCCCACAAGGATGAGGT-3’ |
|  | R: 5’-GATGGCTTTCAGCGTTTCCC-3’ |
| C4BPA | F: 5’-CCAGGCTGTGGAAAGTCTCTG-3’ |
|  | R: 5’-TCGGGGCAGCAAATGATAAAG-3’ |
| SCGB3A1 | F: 5’-TCCGCTGCTGCTTTCTTAGTG-3’ |
|  | R: 5’-GAGCCCTCTATGAGGTGGTTC-3’ |
| FOXA2 | F: 5’-GCGACCCCAAGACCTACAG-3’ |
|  | R: 5’-GGTTCTGCCGGTAGAAGGG-3’ |
| LPCAT1 | F: 5’-ACATCCCGATCTGGGGAACT-3’ |
|  | R: 5’-GGCCACTTTCCGTTGGACT-3’ |
| CXCL17 | F: 5’-AGCAGCCTGAATCCAGGGGTCG-3’ |
|  | R: 5’-GCTGGCAGGCTCTGGAATGCT-3’ |
| TMSB4X | F: 5’-AGACCAGACTTCGCTCGTA-3’ |
|  | R: 5’-CTGCTTGCTTCTCCTGTT-3’ |
| DDX58 | F: 5’-CCTACCTACATCCTGAGCTACAT-3’ |
|  | R: 5’-TCTAGGGCATCCAAAAAGCCA-3’ |
| CCNDBP1 | F: 5’-ACAACTCTGACAACCACAATCAT-3’ |
|  | R: 5’-GCAGGCTTTGGATGCTCTC-3’ |
| Actin | F: 5’-CGGGAAATCGTGCGTGAC-3’ |
|  | R: 5’-CAGGAAGGAAGGCTGGAAG-3’ |

F: Forward primer; R: Reverse primer.
