# Supplementary material for: Suppression subtractive hybridization identified differentially expressed genes in lung adenocarcinoma: ERGIC3 as a novel lung cancer-related gene
Source: BMC Cancer. 2013 Feb 1;13:44. doi: 10.1186/1471-2407-13-44 (PMC3567939; doi:10.1186/1471-2407-13-44)
Supplement: Additional file 2 — Representative differentially expressed genes with identified chromosome locations in the forward-subtracted library of primary lung adenocarcinoma. [file 1471-2407-13-44-S2.doc]

Additional file 2. Representative differentially expressed genes with identiﬁed chromosome locations in the forward-subtracted library of primary lung adenocarcinoma.

| **Accession** | **Definition** | **Cytoband** | **Clone** |
| --- | --- | --- | --- |
| XM_002345305 | Homo sapiens similar to DC48 (LOC100293563) | 1 | 1 |
| NM_002790 | proteasome (prosome, macropain) subunit, alpha type, 5 (PSMA5) | 1p13 | 7 |
| NM_001008218 | amylase, alpha 1B (salivary) (AMY1B) | 1p21 | 2 |
| NM_002353 | tumor-associated calcium signal transducer 2 (TACSTD2) | 1p32-p31 | 3 |
| NM_000147 | fucosidase, alpha-L- 1, tissue (FUCA1) | 1p34 | 2 |
| NM_006004 | ubiquinol-cytochrome c reductase hinge protin (UQCRH) | 1p34.1 | 2 |
| NM_005727 | tetraspanin 1 (TSPAN1) | 1p34.1 | 1 |
| NM_004552 | NADH dehydrogenase (ubiquinone) Fe-S protein 5, (NDUFS5) | 1p34.2-p33 | 7 |
| XR_078216 | similar to cytochrome c oxidase subunit II | 1p36.33 | 5 |
| NM_001144868 | cyclin L2 (CCNL2) | 1p36.33 | 1 |
| XM_002342023 | similar to OK/SW-CL.16 (LOC100288418) | 1p36.33 | 1 |
| NM_001428 | enolase 1, (alpha) (ENO1) | 1p36.3-p36.2 | 4 |
| NM_014624 | S100 calcium binding protein A6 (S100A6) | 1q21 | 1 |
| NM_001025107 | adenosine deaminase, RNA-specific (ADAR) | 1q21.1-q21.2 | 1 |
| NM_003944 | selenium binding protein 1 (SELENBP1) | 1q21-q22 | 2 |
| NG_009898 | roadblock domain containing 3 (ROBLD3) | 1q22 | 5 |
| NM_006469 | influenza virus NS1A binding protein (IVNS1ABP) | 1q25.1-q31.1 | 1 |
| NM_001033056 | glutamate-ammonia ligase (glutamine synthetase) (GLUL) | 1q31 | 2 |
| NM_000715 | complement component 4 binding protein, alpha (C4BPA) | 1q32 | 12 |
| NM_001001552 | LEM domain containing 1 (LEMD1) | 1q32.1 | 3 |
| NM_001083924 | chromosome 1 open reading frame 116 (C1orf116) | 1q32.1-q32.2 | 2 |
| NM_001748 | calpain 2, (m/II) large subunit (CAPN2) | 1q41-q42 | 1 |
| NT_022171 | chromosome 2 genomic contig, GRCh37 reference primary assembly | 2 | 1 |
| XM_002345543 | hypothetical protein LOC100293679 (LOC100293679) | 2 | 3 |
| AB064067 | IGK mRNA for immunoglobulin kappa light chain VLJ region, partial cds, lone:K26 | 2p112 | 1 |
| NM_001143960 | chromosome 2 open reading frame 74 (C2orf74) | 2p15 | 1 |
| NM_002954 | ribosomal protein S27a (RPS27A) | 2p16 | 7 |
| NM_020463 | SMEK homolog 2, suppressor of mek1 (Dictyostelium) (SMEK2) | 2p16.1 | 2 |
| NM_001006946 | syndecan 1 (SDC1) | 2p24.1 | 4 |
| AK290474 | excision repair cross-complementing rodent repair deficiency, complementation group 3 (xeroderma pigmentosum group B complementing) (ERCC3) | 2q21 | 1 |
| NR_002315 | H3 histone, family 3A pseudogene (LOC440926) | 2q31.1 | 1 |
| NM_007315 | signal transducer and activator of | 2q32.2 | 4 |

Additional file 2 (Continued)

| **Accession** | **Definition** | | **Cytoband** | **Clone** |
| --- | --- | --- | --- | --- |
| NM_133631 | transcription 1(STAT1)  roundabout, axon guidance receptor, homolog 1 (Drosophila) (ROBO1) | 3p12 | | 3 |
| NM_002295 | ribosomal protein SA (RPSA) | 3p22.2 | | 4 |
| NM_001034996 | ribosomal protein L14 (RPL14) | 3p22-p21.2 | | 1 |
| NM_015150 | raftlin, lipid raft linker 1 (RFTN1) | 3p25.1-p24.3 | | 1 |
| NM_022149 | melanoma antigen family F, 1 (MAGEF1) | 3q13 | | 1 |
| NM_018266 | transmembrane protein 39A | 3q13.33 | | 1 |
| BC003192 | transmembrane protein 39A, mRNA (cDNA clone IMAGE:3501645), complete cds. | 3q13.33 | | 1 |
| NM_020800 | intraflagellar transport 80 homolog (Chlamydomonas) (IFT80) | 3q25.33 | | 1 |
| NM_000996 | ribosomal protein L35a (RPL35A） | 3q29-qter | | 3 |
| NM_001014446 | OCIA domain containing 2 (OCIAD2) | 4p11 | | 3 |
| NM_015187 | sel-1 suppressor of lin-12-like 3 | 4p15.2 | | 1 |
| NM_024830 | lysophosphatidylcholine acyltransferase 1 (LPCAT1) | 5p15.33 | | 4 |
| NM_001553 | insulin-like growth factor binding protein 7 (IGFBP7) | 4q12 | | 1 |
| NM_001284 | adaptor-related protein complex 3, sigma 1 subunit (AP3S1) | 5q22 | |  |
| NM_080670 | solute carrier family 35, member A4 (SLC35A4) | 5q31.3 | | 5 |
| NM_052863 | secretoglobin, family 3A, member 1 (SCGB3A1) | 5q35-qter | | 2 |
| NT_167248 | chromosome 6 genomic contig, GRCh37 reference assembly alternate locus group ALT_REF_LOCI_6 | 6 | | 1 |
| NM_019111 | major histocompatibility complex, class II, DR alpha (HLA-DRA) | 6p21.3 | | 4 |
| NM_022551 | ribosomal protein S18 | 6p21.3 | | 7 |
| M77774 | Human HLA-B*5401 | 6p21.3 | | 1 |
| NM_001954 | discoidin domain receptor tyrosine kinase 1 (DDR1) | 6p21.3 | | 1 |
| NM_005514 | major histocompatibility complex, class I, B (HLA-B) | 6p21.3 | | 2 |
| BC024269 | major histocompatibility complex, class II, DR beta 1,HLA-DRB1 | 6p21.3 | | 3 |
| NM_001014 | ribosomal protein S10 (RPS10) | 6p21.31 | | 2 |
| NM_001402 | eukaryotic translation elongation factor 1 alpha | 6q14.1 | | 12 |
| NM_018247 | transmembrane protein 30A (TMEM30A) | 6q14.1 | | 1 |
| NM_014320 | heme binding protein 2 (HEBP2) | 6q24 | | 1 |
| NM_001621 | aryl hydrocarbon receptor (AHR) | 7p15 | | 1 |
| NM_019059 | translocase of outer mitochondrial membrane 7 homolog (yeast) (TOMM7) | 7p15.3 | | 1 |
| NM_138446 | Homo sapiens chromosome 7 open reading frame 30 (C7orf30) | 7p15.3 | | 2 |
| NM_176813 | anterior gradient homolog 3 (Xenopus laevis) (AGR3) | 7p21.1 | | 4 |
| NM_014038 | basic leucine zipper and W2 domains 2 | 7p21.1 | | 1 |
|  | (BZW2) |  | |  |
|  |  |  | |  |

Additional file 2 (Continued)

| **Accession** | **Definition** | | **Cytoband** | **Clone** |
| --- | --- | --- | --- | --- |
| NM_006408 | anterior gradient homolog 2 (Xenopus laevis) (AGR2) | 7p21.3 | | 8 |
| NM_002489 | NADH dehydrogenase (ubiquinone) 1 alpha subcomplex, DUFA4) | 7p21.3 | | 1 |
| NM_018890 | ras-related C3 botulinum toxin substrate 1 (rho family, small GTP binding protein Rac1) (RAC1) | 7p22 | | 1 |
| NR_003614 | postmeiotic segregation increased 2-like 2 pseudogene (PMS2L2) | 7q11-q22 | | 1 |
| NM_024636 | STEAP family member 4 (STEAP4) | 7q21.12 | | 1 |
| NM_006234 | polymerase (RNA) II (DNA directed) polypeptide J (POLR2J) | 7q22.1 | | 1 |
| NM_006833 | COP9 constitutive photomorphogenic homolog subunit 6 (COPS6) | 7q22.1 | | 2 |
| NM_145808 | myotrophin (MTPN) | 7q33 | | 1 |
| NM_003143 | single-stranded DNA binding protein 1 (SSBP1) | 7q34 | | 1 |
| NM_001127505 | N-acylsphingosine amidohydrolase (acid ceramidase) 1 (ASAH1) | 8p22-p21.3 | | 1 |
| NM_001023 | ribosomal protein S20 (RPS20) | 8q12 | | 1 |
| NM_001444 | fatty acid binding protein 5 (psoriasis-associated) (FABP5) | 8q21.13 | | 1 |
| NM_014314 | DEAD (Asp-Glu-Ala-Asp) box polypeptide 58 (DDX58) | 9p12 | | 1 |
| NM_012203 | glyoxylate reductase/hydroxypyruvate reductase (GRHPR) | 9q12 | | 1 |
| NM_000976 | ribosomal protein L12 (RPL12) | 9q34 | | 1 |
| NM_007209 | ribosomal protein L35 (RPL35) | 9q34.1 | | 8 |
| NM_001128309 | tetraspanin 14 (TSPAN14) | 10q23.1 | | 1 |
| NM_001127227 | CD59 molecule, complement regulatory protein (CD59) | 11p13 | | 1 |
| NM_001017 | ribosomal protein S13 (RPS13) | 11p15 | | 1 |
| NM_001004 | ribosomal protein, large, P2 (RPLP2) | 11p15.5-p15.4 | | 1 |
| NM_002032 | ferritin, heavy polypeptide 1 (FTH1) | 11q13 | | 4 |
| NM_000852 | glutathione S-transferase pi 1 (GSTP1) | 11q13 | | 2 |
| NM_001143985 | barrier to autointegration factor 1 (BANF1) | 11q13.1 | | 1 |
| NM_170738 | mitochondrial ribosomal protein L11 (MRPL11) | 11q13.3 | | 1 |
| NM_016731 | folate receptor 1 (adult) (FOLR1) | 11q13.3-q14.1 | | 3 |
| NM_016565 | coiled-coil-helix-coiled-coil-helix domain containing 8 (CHCHD8) | 11q13.4 | | 1 |
| NM_001814 | cathepsin C (CTSC) | 11q14.1-q14.3 | | 1 |
| NM_033306 | caspase 4, apoptosis-related cysteine peptidase (CASP4) | 11q22.2-q22.3 | | 6 |
| NM_004585 | retinoic acid receptor responder (tazarotene induced) 3 (RARRES3) | 11q23 | | 4 |
| NM_000019 | acetyl-Coenzyme A acetyltransferase 1 (ACAT1) | 11q22.3-q23. | | 1 |
| NM_001028 | ribosomal protein S25 (RPS25) | 11q23. | | 6 |
| BC004294 | similar to protein kinase, cAMP | 11q23.3 | | 1 |
|  | dependent regulatory, type I beta (cDNA clone IMAGE:3349336) |  | |  |
|  |  |  | |  |
|  |  |  | |  |

Additional file 2 (Continued)

| **Accession** | **Definition** | | **Cytoband** | **Clone** |
| --- | --- | --- | --- | --- |
| NR_024516 | amyloid beta (A4) precursor-like protein 2 (APLP2) | 11q23-q25; 11q24 | | 1 |
| NM_001175 | Rho GDP dissociation inhibitor (GDI) beta (ARHGDIB) | 12p12.3 | | 1 |
| NM_001769 | CD9 molecule (CD9) | 12p13.3 | | 2 |
| NM_003153 | signal transducer and activator of transcription 6, interleukin-4 induced (STAT6) | 12q13 | | 1 |
| NM_147190 | LAG1 homolog, ceramide synthase 5 (LASS5) | 12q13.12 | | 1 |
| NM_001731 | B-cell translocation gene 1, anti-proliferative (BTG1) | 12q22 | | 1 |
| NM_006253 | protein kinase, AMP-activated, beta 1 non-catalytic subunit (PRKAB1) | 12q24.1 | | 2 |
| NM_003299 | heat shock protein 90kDa beta (Grp94), member 1 (HSP90B1) | 12q24.2-q24.3 | | 1 |
| NM_005870 | Sin3A-associated protein, 18kDa (SAP18) | 13q12.11 | | 1 |
| XM_002344734 | PREDICTED: Homo sapiens similar to ribosomal protein L13a (LOC100293761) | 13q14.3 | | 1 |
| AK304469 | cDNA FLJ59083 complete cds, highly similar to Ig gamma-1 chain C region. | 14 | | 1 |
| XM_002344756 | hypothetical protein LOC100292323 | 14q11.2 | | 1 |
| NM_002933 | ribonuclease, RNase A family, 1(RNASE1) | 14q11.2 | | 6 |
| NM_002818 | proteasome (prosome, macropain) activator subunit 2 (PA28 beta) (PSME2) | 14q11.2 | | 1 |
| NM_006432 | Niemann-Pick disease, type C2 (NPC2) | 14q24.3 | | 4 |
| AK097572 | sapiens cDNA FLJ40253 fis, clone TESTI2024419 | 14q32.33 | | 4 |
| NG_001019 | immunoglobulin heavy locus (IGH@) on chromosome 14 | 14q32.33 | | 1 |
| NM_037370 | cyclin D-type binding-protein 1 (CCNDBP1) | 15q14-q15 | | 1 |
| NM_001030009 | ribosomal protein S15a | 16p | | 9 |
| NM_001130007 | G1 to S phase transition 1 (GSPT1) | 16p13.1 | | 5 |
| NM_001040146 | chromosome transmission fidelity factor 8 homolog (S. cerevisiae) transcript variant 4 (CHTF8） | 16q22.1 | | 3 |
| NM_001861 | cytochrome c oxidase subunit IV isoform 1 (COX4I1) | 16q22-qter | | 1 |
| NM_015510 | dehydrogenase/reductase (SDR family) member 7B (DHRS7B) | 17p12 | | 1 |
| NM_002798 | proteasome (prosome, macropain) subunit, beta type, 6 (PSMB6) | 17p13 | | 1 |
| NM_001416 | eukaryotic translation initiation factor 4A, isoform 1 (EIF4A1) | 17p13 | | 1 |
| NM_033389 | slingshot homolog 2 (Drosophila) (SSH2) | 17q11.2 | | 1 |
| NM_000981 | ribosomal protein L19 (RPL19) | 17q11.2-q12 | | 1 |
| NM_003734 | amine oxidase, copper containing 3 | 17q21 | | 1 |
|  | (vascular adhesion protein 1) (AOC3) |  | |  |
| NM_001144001 | SEC14-like 1 (S. cerevisiae) (SEC14L1) | 17q25.1-q25.2 | | 1 |
| NM_033280 | SEC11 homolog C (S. cerevisiae) (SEC11C) | 18q21.32 | | 1 |

Additional file 2 (Continued)

| **Accession** | **Definition** | | **Cytoband** | **Clone** |
| --- | --- | --- | --- | --- |
| NM_000980 | ribosomal protein L18a (RPL18A) | 19p13 | | 8 |
| NM_003333 | ubiquitin A-52 residue ribosomal protein fusion product 1 (UBA52) | 19p13.1-p12 | | 2 |
| NR_023313 | cold inducible RNA binding protein (CIRBP) | 19p13.3 | | 3 |
| NM_001018 | ribosomal protein S15 (RPS15) | 19p13.3 | | 1 |
| NM_000064 | complement component 3 (C3) | 19p13.3-p13.2 | | 1 |
| NM_021102 | serine peptidase inhibitor, Kunitz type, 2 (SPINT2) | 19q13.1 | | 4 |
| NM_002483 | carcinoembryonic antigen-related cell adhesion molecule 6 (non-specific cross reacting antigen) (CEACAM6) | 19q13.2 | | 3 |
| NM_198477 | chemokine (C-X-C motif) ligand 17 (CXCL17) | 19q13.2 | | 7 |
| NM_001022 | ribosomal protein S19 (RPS19) | 19q13.2 | | 2 |
| NM_012423 | ribosomal protein L13a (RPL13A) | 19q13.3 | | 2 |
| NM_004851 | napsin A aspartic peptidase (NAPSA) | 19q13.33 | | 4 |
| NM_015629 | PRP31 pre-mRNA processing factor 31 homolog (S. cerevisiae) (PRPF31) | 19q13.42 | | 2 |
| XM_002345401 | forkhead box A2 (FOXA2) | 20p11 | | 1 |
| XR_078903 | hypothetical LOC100292556 (LOC100292556) | 20p11.22 | | 1 |
| NM_015966 | ERGIC and golgi 3 (ERGIC3) | 20pter-q12 | | 1 |
| NM_005534 | interferon gamma receptor 2 (IFNGR2) | 21q22.11 | | 1 |
| NM_080748 | reactive oxygen species modulator 1 (ROMO1) | 20q11.22 | | 1 |
| NM_001697 | ATP synthase, H^+^ transporting, mitochondrial F1 complex, O subunit (ATP5O) | 21q22.1-q22.2; 21q22.11 | | 1 |
| NM_004965 | high-mobility group nucleosome binding domain 1 (HMGN1) | 21q22.2 | | 1 |
| XM_002831180 | PREDICTED: Pongo abelii RING-box protein 1-like | 22 | | 1 |
| X57819 | rearranged immunoglobulin lambda light chain | 22 | | 1 |
| AC239875 | FOSMID clone COR02-DD0002RVINU  _H24 from chromosome 22 | 22 | | 1 |
| XM_002348096 | hypothetical protein LOC100287927 (LOC100287927) | 22q11.22 | | 6 |
| NM_005318 | H1 histone family, member 0 (H1F0) | 22q13.1 | | 1 |
| NM_182810 | activating transcription factor 4 (ATF4) | 22q13.1 | | 1 |
| NM_014248 | ring-box 1 (RBX1) | 22q13.2 | | 1 |
| NM_001005333 | melanoma antigen family D, 1(MAGED1) | Xp11.23 | | 4 |
| NM_000284 | pyruvate dehydrogenase (lipoamide) alpha 1 (PDHA1) | Xp22.2-p22.1 | | 1 |
| NM_018486 | Histone deacetylase 8 (HDAC8) | Xq13 | | 2 |
| AK301740 | phosphoglycerate kinase 1 (PGK1) | Xq13 | | 1 |
| NM_001007 | ribosomal protein S4, X-linked (RPS4X) | Xq13.1 | | 9 |
| NM_001866.2 | cytochrome c oxidase subunit VIIb (COX7B) | Xq21.1 | | 1 |
| NM_021109 | thymosin beta 4, X-linked (TMSB4X) | Xq21.3-q22 | | 3 |
| NM_021029 | ribosomal protein L36a (RPL36A) | Xq22.1 | | 4 |

Additional file 2 (Continued)

| **Accession** | **Definition** | | **Cytoband** | **Clone** |
| --- | --- | --- | --- | --- |
| NM_006667 | progesterone receptor membrane component 1 (PGRMC1) | Xq22-q24 | | 1 |
| NM_006013 | ribosomal protein L10 (RPL10) | Xq28 | | 5 |
| NC_001807.4 | Homo sapiens mitochondrion | MT | | 1 |
| XR_078993.1 | similar to DC24 (LOC100293090) | MT | | 1 |
| BG758779 | 602713139F1 NIH_MGC_48 Homo | Unknown | | 2 |
|  | sapiens cDNA clone |  | |  |
| BC021036 | actin, gamma 1 | Unknown | | 1 |
| HM436820 | isolate Ece mitochondrion, complete genome. | Unknown | | 1 |
| HQ154135 | haplogroup K2A2 mitochondrion | Unknown | | 3 |
| XM_002346250 | similar to HLA class II histocompatibility antigen, DRB1-7 beta chain (LOC100294036) | Unknown | | 2 |
| NM_005567 | Homo sapiens lectin, galactoside-binding, soluble, 3 binding protein (LGALS3BP) | Unknown | | 1 |
| AB289328 | mRNA for immunoglobulin light chain, partial cds, clone: Pf25-L | Unknown | | 3 |
| EU599327 | clone AL-184 immunoglobulin light chain mRNA, partial cds | Unknown | | 1 |
| AB064207 | IGL mRNA for immunoglobulin lambda light chain VLJ region, partial cds, clone:L67 | Unknown | | 5 |
| AB064127 | IGK mRNA for immunoglobulin kappa light chain VLJ region, partial cds, lone:K86 | Unknown | | 1 |
| GQ214526 | haplotype PB036 mitochondrion | Unknown | | 1 |
| XM_002348257 | similar to immunoglobulin lambda-like polypeptide 1 (LOC100294459) | Unknown | | 2 |
| AB289329 | mRNA for immunoglobulin light chain, partial cds, clone: Pf143-L | Unknown | | 1 |

Note: Clone is the number of times each clone was identiﬁed in SSH analysis. Accession, definition, and cytoband were assigned using the NCBI database.
