# Supplementary material for: Suppression subtractive hybridization identified differentially expressed genes in lung adenocarcinoma: ERGIC3 as a novel lung cancer-related gene
Source: BMC Cancer. 2013 Feb 1;13:44. doi: 10.1186/1471-2407-13-44 (PMC3567939; doi:10.1186/1471-2407-13-44)
Supplement: Additional file 3 — Representative differentially expressed genes with identified chromosome locations in the reverse-subtracted library of lung adenocarcinoma. [file 1471-2407-13-44-S3.doc]

Additional file 3. Representative differentially expressed genes with identiﬁed chromosome locations in the reverse-subtracted library of lung adenocarcinoma.

| **Accession** | | **Definition** | | **Cytoband** | | **Clone** |
| --- | --- | --- | --- | --- | --- | --- |
| NM_001400 | sphingosine-1-phosphate receptor 1 (S1PR1) | | 1p21 | | 1 | |
| NM_002038 | interferon, alpha-inducible protein 6 (IFI6) | | 1p35 | | 1 | |
| NM_173452 | ficolin (collagen/fibrinogen domain containing)  3 (Hakata antigen) (FCN3) | | 1p36.11 | | 2 | |
| NM_014624 | S100 calcium binding protein A6 (S100A6) | | 1q21 | | 1 | |
| NM_001043353 | tropomyosin 3 (TPM3) | | 1q21.2 | | 9 | |
| NM_139119 | YY1 associated protein 1 (YY1AP1) | | 1q22 | | 1 | |
| NM_148964 | cathepsin E (CTSE) | | 1q31 | | 2 | |
| NM_020216 | arginyl aminopeptidase (aminopeptidase B) (RNPEP) | | 1q32 | | 1 | |
| NM_014713 | lysosomal protein transmembrane 4 alpha (LAPTM4A) | | 2p24.1 | | 1 | |
| NM_207328 | hypothetical protein LOC150763 | | 2q11.1 | | 1 | |
| NM_004657 | serum deprivation response (phosphatidylserine binding protein) (SDPR) | | 2q32-q33 | | 1 | |
| NM_032495 | HOP homeobox (HOPX), transcript variant 1 | | 4q11-q12 | | 2 | |
| NM_001042734 | SEC24 family, member B (S. cerevisiae) (SEC24B) | | 4q25 | | 1 | |
| NM_002084 | glutathione peroxidase 3 (plasma) (GPX3) | | 5q23 | | 1 | |
| NM_019111 | major histocompatibility complex, class II, DR alpha (HLA-DRA） | | 6p21.3 | | 2 | |
| BC024269 | major histocompatibility complex, class II, DR beta1 | | 6p21.3 | | 6 | |
| NM_014341 | mitochondrial carrier homolog 1 (C. elegans) (MTCH1) | | 6pter-p24.1 | | 1 | |
| NM_002356 | myristoylated alanine-rich protein kinase C substrate (MARCKS) | | 6q22.2 | | 1 | |
| NM_003730 | ribonuclease T2 (RNASET2) | | 6q27 | | 1 | |
| NM_002793 | proteasome (prosome, macropain) subunit, beta type, 1 (PSMB1) | | 6q27 | | 2 | |
| NM_001753 | caveolin 1, caveolae protein, 22kDa (CAV1) | | 7q31.1 | | 1 | |
| NM_005195 | CCAAT/enhancer binding protein (C/EBP), delta (CEBPD) | | 8p11.2-p11.1 | | 2 | |
| NM_033439 | interleukin 33 (IL33) | | 9p24.1 | | 1 | |
| NM_001093770 | surfactant protein A1 (SFTPA1) | | 10q22.3 | | 7 | |
| NM_001098668 | surfactant protein A2 (SFTPA2) | | 10q22.3 | | 7 | |
| NM_005736 | ARP1 actin-related protein 1 homolog A, centractin alpha (yeast) (ACTR1A) | | 10q24.32 | | 1 | |
| NM_001548 | interferon-induced protein with tetratricopeptide  repeats 1 (IFIT1) | | 10q25-q26 | | 1 | |
| NM_001144062 | coatomer protein complex, subunit beta 1 (COPB1) | | 11p15.2 | | 1 | |
| NM_018222 | parvin, alpha (PARVA) | | 11p15.3 | | 1 | |
| NM_021034 | interferon induced transmembrane protein 3 (1-8U)  (IFITM3) | | 11p15.5 | | 1 | |
| NM_001293 | chloride channel, nucleotide-sensitive, 1A (CLNS1A) | | 11q13.5-q14 | | 3 | |
| NM_006931 | solute carrier family 2 (facilitated glucose transporter), member 3 (SLC2A3) | | 12p13. | | 1 | |
| NM_016511 | C-type lectin domain family 1, member A (CLEC1A) | | 12p13.2 | | 6 | |
| NM_001769 | CD9 molecule (CD9) | | 12p13.3 | | 4 | |

Additional file 3 (Continued)

| **Accession** | | **Definition** | **Cytoband** | | **Clone** | |
| --- | --- | --- | --- | --- | --- | --- |
| NM_014505 | potassium large conductance calcium-activated channel, subfamily M, beta member 4 (KCNMB4) | | | 12q | | 4 |
| NM_014033 | methyltransferase like 7A (METTL7A) | | | 12q13.12 | | 3 |
| NM_153377 | leucine-rich repeats and immunoglobulin-like domains 3 (LRIG3) | | | 12q14.1 | | 7 |
| NM_001113202 | nascent polypeptide-associated complex alpha subunit (NACA) | | | 12q23-q24.1 | | 2 |
| NM_002408 | mannosyl(alpha-1,6-)-glycoprotein beta-1,2-N- acetylglucosaminyltransferase (MGAT2) | | | 14q21 | | 1 |
| NM_004048 | beta-2-microglobulin (B2M) | | | 15q21-q22.2 | | 1 |
| NM_001018020 | tropomyosin 1 (alpha) (TPM1) | | | 15q22.1 | | 1 |
| NM_003870 | IQ motif containing GTPase activating protein 1 (IQGAP1) | | | 15q26.1 | | 1 |
| NM_001424 | epithelial membrane protein 2 (EMP2) | | | 16p13.2 | | 1 |
| NM_006985 | nuclear pore complex interacting protein (NPIP) | | | 16p13-p11 | | 5 |
| NM_012106 | ADP-ribosylation factor-like 2 binding protein (ARL2BP) | | | 16q13  continued | | 2 |
| NM_001605 | alanyl-tRNA synthetase (AARS) | | | 16q22 | | 3 |
| NM_001861 | cytochrome c oxidase subunit IV isoform 1 (COX4I1) | | | 16q22-qter | | 5 |
| NM_002661 | phospholipase C, gamma 2 (phosphatidylinositol-specific) (PLCG2) | | | 16q24.1 | | 2 |
| NM_018286 | transmembrane protein 100 (TMEM100) | | | 17q22 | | 5 |
| NM_000717 | carbonic anhydrase IV (CA4) | | | 17q23 | | 2 |
| NM_006145 | DnaJ (Hsp40) homolog, subfamily B, member 1 (DNAJB1) | | | 19p13.2 | | 3 |
| NM_012423 | ribosomal protein L13a (RPL13A) | | | 19q13.3 | | 1 |
| NM_001002836 | Homo sapiens zinc finger protein 787 (ZNF787) | | | 19q13.43 | | 1 |
| NM_198391 | fibronectin leucine rich transmembrane protein 3 (FLRT3) | | | 20p11 | | 5 |
| NM_007367 | RNA binding protein, autoantigenic (hnRNP-associated with lethal yellow homolog (mouse)) (RALY) | | | 20q11.21-q11.23 | | 1 |
| NM_000362 | TIMP metallopeptidase inhibitor 3 (TIMP3) | | | 22q12.1-q13.2 | | 1 |
| NC_001807 | mitochondrion, complete genome | | | MT | | 2 |
| NM_021109 | thymosin beta 4, X-linked (TMSB4X) | | | Xq21.3-q22 | | 1 |
| NT_016354.19 | chromosome 4 genomic contig, GRCh37 reference primary assembly | | | unknown | | 1 |

Note: Clone is the number of times each clone was identiﬁed in SSH analysis. Accession, definition, and cytoband were assigned using the NCBI database.
