# Supplementary material for: Suppression subtractive hybridization identified differentially expressed genes in lung adenocarcinoma: ERGIC3 as a novel lung cancer-related gene
Source: BMC Cancer. 2013 Feb 1;13:44. doi: 10.1186/1471-2407-13-44 (PMC3567939; doi:10.1186/1471-2407-13-44)
Supplement: Additional file 4 — The genes appeared twice or three times in the different forward- subtracted libraries of lung cancer by suppression subtractive hybridization. [file 1471-2407-13-44-S4.doc]

**Additional file 4. The genes appeared twice or three times in the different forward- subtracted libraries of lung cancer by suppression subtractive hybridization.**

| Gene | Frequency of occurrence | References |
| --- | --- | --- |
| AHR | 2 | Liu *et al.* (2007)/Our FSL |
| AKR1B10 | 2 | Liu *et al.* (2007)/Petroziello *et al.* (2004) |
| APLP2 | 2 | Liu *et al.* (2007)/Our FSL |
| ARHGEF7 | 2 | Liu *et al.* (2007)/Bangur *et al.* (2002) |
| B2M | 2 | Sun *et al.* (2004)/Liu *et al.* (2007) |
| GNB1 | 2 | Difilippantonio *et al.* (2003)/Bangur *et al.* (2002) |
| CANX | 2 | Petroziello *et al.* (2004)/Liu *et al.* (2007) |
| CAST | 2 | Petroziello *et al.* (2004)/Bangur *et al.* (2002) |
| CD59 | 2 | Petroziello *et al.* (2004)/Our FSL |
| CLDN1 | 2 | Bangur *et al.* (2002)/Petroziello *et al.* (2004) |
| COX4I1 | 2 | Sun *et al.* (2004)/Our FSL |
| CRMP1 | 2 | Difilippantonio *et al.* (2003)/Bangur *et al.* (2002) |
| CTSC | 2 | Difilippantonio *et al.* (2003)/Our FSL |
| CYP1B1 | 2 | Petroziello *et al.* (2004)/Liu *et al.* (2007) |
| EIF3S10 | 2 | Petroziello *et al.* (2004)/Sun *et al.* (2004) |
| ENO1 | 2 | Petroziello *et al.* (2004)/Our FSL |
| FN1 | 2 | Sun *et al.* (2004)/Liu *et al.* (2007) |
| GLTP | 2 | Sun *et al.* (2004)/Liu *et al. (*2007) |
| GLUL | 2 | Sun *et al.* (2004)/Our FSL |
| GNAS | 2 | Sun *et al.* (2004)/Liu *et al.* (2007) |
| HSP90B1 | 2 | Petroziello *et al.* (2004)/Our FSL |
| KPNA2 | 2 | Petroziello *et al.* (2004)/ Difilippantonio *et al.* (2003) |
| LDHA | 2 | Petroziello *et al.* (2004)/Bangur *et al.* (2002) |
| MAGEA6 | 2 | Difilippantonio *et al.* (2003)/Petroziello *et al.* (2004) |
| PC4 | 2 | Petroziello *et al.* (2004)/Liu *et al.* (2007) |
| PGK1 | 2 | Petroziello *et al.* (2004)/Our FSL |
| PTTG1 | 2 | Petroziello *et al.* (2004)/Bangur *et al.* (2002) |
| RAC1 | 2 | Liu *et al.* (2007)/Our FSL |
| RBX1 | 2 | Liu *et al.* (2007)/Our FSL |
| RPL10 | 2 | Liu *et al.* (2007)/Our FSL |
| RPL19 | 2 | Petroziello *et al.* (2004)/Our FSL |
| RPL35 | 2 | Liu *et al.* (2007)/Our FSL |
| RPS10 | 2 | Petroziello *et al*. (2004)/Our FSL |
| RPS13 | 2 | Petroziello *et al.* (2004)/Our FSL |
| RPS20 | 2 | Liu *et al.* (2007)/Our FSL |
| RPS27A | 2 | Liu *et al.* (2007)/Our FSL |
| SDF2 | 2 | Difilippantonio *et al.* (2003)/ Liu *et al.* (2007) |

Additional file 4 (Continued).

| Gene | Frequency of occurrence | References |
| --- | --- | --- |
| SET | 2 | Petroziello *et al.* (2004)/Liu *et al.* (2007) |
| SNRPG | 2 | Difilippantonio *et al.* (2003)/ Liu *et al.* (2007) |
| SPP1 | 2 | Petroziello *et al.* (2004)/ Liu *et al.* (2007) |
| TOP2A | 2 | Petroziello *et al.* (2004)/Bangur *et al*. (2002) |
| TXNRD1 | 2 | Petroziello *et al.* (2004)/Liu *et al.* (2007) |
| EEF1A1 | 3 | Petroziello *et al.* (2004)/Liu *et al.* (2007)/Our FSL |
| FTH1 | 3 | Petroziello *et al.* (2004)/Liu *et al.* (2007)/Our FSL |
| GSTP1 | 3 | Petroziello *et al.* (2004)/Difilippantonio *et al.* (2003)/Our FSL |
| STAT1 | 3 | Liu *et al.* (2007)/Bangur *et al.* (2002)/Our FSL |

FSL: forward-subtracted library.
