# Supplementary material for: Suppression subtractive hybridization identified differentially expressed genes in lung adenocarcinoma: ERGIC3 as a novel lung cancer-related gene
Source: BMC Cancer. 2013 Feb 1;13:44. doi: 10.1186/1471-2407-13-44 (PMC3567939; doi:10.1186/1471-2407-13-44)
Supplement: Additional file 5 — The genes appeared twice in the different reverse-subtracted libraries of lung cancer by suppression subtractive hybridization. [file 1471-2407-13-44-S5.doc]

**Additional file 5. The genes appeared twice in the different reverse-subtracted libraries of lung cancer by suppression subtractive hybridization.**

| Gene | Frequency of occurrence | References |
| --- | --- | --- |
| ANXA8 | 2 | Difilippantonio *et al.* (2003)/Sun *et al.* (2004) |
| CAV1 | 2 | Difilippantonio *et al.* (2003)/Our RSL |
| CEBPD | 2 | Difilippantonio *et al.* (2003)/Our RSL |
| GPX3 | 2 | Difilippantonio *et al.* (2003)/Our RSL |
| TPM3 | 2 | Sun *et al.* (2004)/Our RSL |
| NACA | 2 | Sun *et al.* (2004)/Our RSL |

RSL: reverse-subtracted library.
